# Dietary Patterns and Cardiovascular Disease-Related Risks in Chinese Older Adults

**DOI:** 10.3389/fpubh.2013.00048

**Published:** 2013-11-01

**Authors:** Jing Sun, Nicholas Buys, Shuying Shen

**Affiliations:** ^1^School of Public Health, Griffith Health Institute, Griffith University, Gold Coast, QLD, Australia; ^2^Chang Shu Health Inspection Institute, Chang Shu, China

**Keywords:** dietary pattern, cardiovascular disease, hypertension, obesity, metabolic syndrome

## Abstract

Studies of Western populations demonstrate a relationship between dietary patterns and cardiovascular-related risk factors. Similar research regarding Chinese populations is limited. This study explored the dietary patterns of Chinese older adults and their association with cardiovascular-related risk factors, including hypertension, obesity, and metabolic syndrome. Data were collected using a 34-item Chinese food frequency questionnaire from 750 randomly selected older adults aged 50–88 who participated in the study in 2012. Factor analysis revealed four dietary patterns: a “traditional food pattern,” consisting of vegetable, fruit, rice, pork, and fish; a “fast and processed food pattern” consisting of fast or processed food products, sugar, and confectionery; a “soybean, grain, and flour food pattern”; and a “dairy, animal liver, and other animal food pattern.” These patterns explained 17.48, 9.52, 5.51, and 4.80% of the variances in food intake, respectively. This study suggests that specific dietary patterns are evident in Chinese older adults. Moderate intake of “traditional Chinese food” is associated with decreased blood pressure and cholesterol level. A dietary pattern rich in soybeans, grains, potatoes, and flour is associated with reduced metabolic factors including reduced triglycerides, fasting glucose, waist circumference, and waist–hip ratio, and a high level of dairy, animal liver, and other animal intake food pattern is associated with increased level of Body Mass Index. In conclusion, this study revealed identifiable dietary patterns among Chinese older adults that are significantly related to blood pressure and metabolic biomarkers. Further study using prospective cohort or intervention study should be used to confirm the association between dietary patterns and blood pressure and metabolic factors.

## Introduction

The prevalence of hypertension is increasing and constitutes a major public health issue in China (1). According to the 2002 Chinese national health survey, 28.6% of the population aged 35 or above has hypertension, with the percentage increasing to 48.8% for those aged 65 or above (2). Hypertension is associated with leading causes of morbidity and mortality, including metabolic syndrome, diabetes, stroke, heart attack, and kidney failure (3). There is substantial evidence that individuals with different food intake patterns and exercise levels have different levels of cardiovascular risk factors such as high blood pressure, high levels of serum cholesterols, and low-density lipoprotein (LDL)-cholesterol (4–6). For example, people who exercise and consume healthy food have a relatively low risk of demonstrating cardiovascular disease risk factors (7) and ischemic heart disease mortality (8). Most of these studies have been on Western populations, research in this area on Chinese older adult populations is limited.

The relationship between hypertension, diet, and exercise also exists in the older adult population. Despite the importance of nutrition for health, many older adults’ food intake patterns are inconsistent with dietary guidelines. For example, studies from the United States, Europe, and Australia have demonstrated that older adults tend to have poor intakes of fruit, vegetables, dairy products, and whole grains, and a higher than desirable consumption of fatty food and fast food (9). Consequently, many older people fall short of achieving optimal nutrient intakes for good health. These unhealthy dietary patterns are associated with high risk of obesity (10) metabolic syndrome (11, 12), glucose abnormality (13), and cardiovascular disease (14).

Dietary patterns that conform with Western dietary guidelines are consistently associated with a lower risk of mortality (9, 15). A high consumption of vegetables and fruit is associated with decreased blood pressure in adults (16, 17), and higher than recommended food intake in fats and meat products is associated with being overweight and obese (18, 19), although this association is less clear in older adults. It has been established that a high risk for metabolic syndrome, diabetes, cardiovascular disease, and stroke can be mediated or modulated through effects on blood pressure, central body weight and obesity, fasting glucose, lipoprotein, and triglycerides (20). However, the relationship of dietary patterns to these biomarkers in Chinese older adults is unclear.

Several studies support the need for further research into dietary patterns and their effect on morbidity and mortality in older adults (21, 22). Dietary patterns have been shown to reflect variations in nutrient intake (13, 20, 23). Individuals consume nutrients through a combination of food items; therefore, focusing on one specific dietary component is often inappropriate for determining the interactive effects among nutrients that might affect health outcomes in older adults. A focus on the complete dietary pattern of individuals is essential to capture complex diet behaviors and the potential relationship between nutrients and health outcomes (13, 14, 23).

Some studies (24, 25) have found strong socioeconomic gradients in identified dietary patterns. For example, higher incomes and education levels are usually found to be associated with higher scores on the Healthy Eating Index, the Mediterranean Diet Score, and the Healthy Dietary Pattern (25). Other lifestyle factors such as smoking, alcohol consumption, and physical inactivity are reportedly related to both unhealthy dietary patterns (25) and mortality risks (26). The effect of cigarette smoking on weight status and blood pressure has been less studied in older people than it has been studied in adulthood, in which smoking has been found to be associated with increased arterial stiffness (27) and dyslipidemia (28–30). Physical inactivity is an independent risk factor for cardiovascular disease in older adults and has been found to be associated with high blood pressure (31, 32), high serum cholesterol, and lipid cholesterol, as well as glucose intolerance (33). The effects of smoking, alcohol consumption, and physical inactivity on the association between dietary patterns and cardiovascular risk factors have not been established in older people in China.

Given the lack of research on dietary patterns and cardiovascular risk factors in Chinese older adults, the aim of this study was to investigate the relationship between dietary habits and cardiovascular risk factors such as elevated blood pressure, metabolic syndrome, weight levels, and obesity in this population. The effects of socio-demographic factors, smoking, alcohol consumption, and physical inactivity on the relationship between dietary and cardiovascular risk factors were also examined. The following hypothesis was therefore proposed: there is relationship between dietary pattern and cardiovascular risk factors including elevated blood pressure, metabolic syndrome, and obesity in Chinese older adults.

## Materials and Methods

### Study design, setting, participants, and procedure

Participants were randomly selected from people registered for a health check with the Changshu Centre for Disease Control and Prevention (CDC) in 2012. The study population consisted of Chinese older people aged 45–80 years in Changshu, Jiangsu Province. Changshu is situated in Southern China. Changshu has a gross domestic product (GDP) of over 4.80 trillion yuan (US$759 billion), placing it second highest in a ranking of all Chinese provinces and municipal cities (34). Changshu in Jiangsu Province was selected as the study site because of its representation of small to medium size cities where most Chinese people reside. It also has well developed economic status in China, which has resulted in dramatic changes in the lifestyle, dietary, and disease patterns of its residents in recent years. Chronic, non-communicable diseases now account for an estimated 80% of total deaths and 70% of disability-adjusted life-years lost in Jiangsu province (35), making it exemplars of this change. To be included in the study participants had to be aged 50 or older, and live within 50 km of a metropolitan area. People who could not provide written consent to the study, had neurological impairments, severe mental illness, stroke, or heart failure were excluded from the study. Of the 750 eligible participants, 550 (73.3%) gave written, informed consent for involvement in the study. This was sufficient to meet the minimum sample size requirement of 143 participants. Participants completed both the Food Frequency Questionnaire (FFQ) and biomarker tests, which were taken by qualified physicians.

### Diet- and activity-related questionnaire

The FFQ was developed as part of this study. The 24-h dietary recall method was used to collect food intake over two weekdays and one weekend on. Pictures were presented of 50 common foods in Chinese dishes/utensils in a range of sizes; the participants were asked to indicate portion/sizes consumed and how often they consumed the food (daily, weekly, or monthly). From this data, 34 frequently consumed items (90% of participants have chosen) were selected and grouped as follows: rice, flour, grains, beans, soybeans, soybean products, nuts including peanuts and sunflower seeds, deep-color vegetables, light-color vegetables, edible fungi and algae (such as mushrooms, agaric, and kelp), fruit, pork, poultry, seafood, beef/lamb/other red meat, organ meat, processed meat (such as ham, luncheon meat, canned meat), eggs, milk and yogurt, milk powder and cheese, confectionery, fast food, sugar, puffed food, and pastries. The amount of consumption of each food item was calculated individually per day per person.

Exploratory factor analysis extracted four dietary patterns from the 34-items. These patterns are best described as: (1) a “traditional food pattern” consisting of pork, poultry, fish and prawns, eggs, fruit, dark-color vegetables, light-color vegetables, rice, water, yogurt, fungi, peanuts, and sunflower seeds, pastries; (2) a “processed and fast food pattern,” high in fats and sugars, consisting of meat snacks, puffed food, fast food, confectionery, carbonated drinks, and fruit and vegetable products; (3) a “soybean, grains, and flour food pattern” consisting of beans, grains, potatoes, soybeans, flours, and soybean products; and (4) a “milk products, other animal meats and animal organs pattern” consisting of animal liver, animal blood, other animal meats, commercial fruit and vegetable juices, cheese, fruit juices, fresh milk, and milk drinks. These patterns explained 17.48, 9.52, 5.57, and 4.80% of the variance, respectively, comprising 37.37% of the total variance. The item milk powder had low factor loading. This item was deleted from the 34-item FFQ. Cronbach’s alpha coefficients of 0.76 indicated a reasonable level of inter-item reliability for the questionnaire (0.69 for the traditional food pattern; 0.65 for the processed and fast food pattern; 0.73 for the soybeans, grains, and flour food pattern, and 0.63 for the milk products, animal liver, and other animal meat pattern, respectively). Pearson’s correlation coefficients between the scores of the four patterns ranged from 0.13 to 0.27, suggesting that these four food patterns are discrete and therefore independent of each other.

### Anthropometry measures

The participants, wearing light, indoor clothing without shoes, were weighed to the nearest 0.1 kg and measured to the nearest 0.1 cm in height. BMI was calculated as weight (kilogram)/height (meter)^2^. The China Centre for Disease Control and Prevention (China CDC) and the Ministry of Health have adopted the World Diabetes Federation criteria (36) based on the BMI, and define “overweight” as 24.0–29.9 kg/m^2^ and obesity as 30 and more than 30 kg/m^2^.

Waist and hip girth (centimeter) were measured with an anthropometric tape over light clothing. Waist girth was measured at the minimum circumference between the iliac crest and the ribcage, and hip girth at the maximum width over the greater trochanters. The waist–hip ratio was then calculated as waist divided by hip measurements. Abdominal obesity was defined as waist circumference (WC), the maximum width over the greater abdomen, based on the World Diabetes Federation standard (36).

Systolic blood pressure (SBP) and diastolic blood pressure (DBP) were measured using an inflatable cuff wrapped around the upper arm (not the forearm or wrist) and attached to an electronic monitor that gave a digital readout of the BP (millimeter of mercury) and pulse. The average scores of SBP and DBP based on three measurements were used. The criteria for elevated BP are SBP >130 mmHg and DBP >85 mmHg, based on the International Diabetes Federation (IDF) and China CDC criteria (36).

### Measurement of metabolic abnormalities

Metabolic syndrome was based on the updated IDF criteria (36). To be defined as having metabolic syndrome, a person must have central obesity (defined as WC ≥90 cm for Chinese men and ≥80 cm for Chinese women, plus any two of the following four factors: (1) raised TG level: ≥150 mg/dL (1.7 mmol/L); (2) reduced HDL cholesterol: <40 mg/dL (1.03 mmol/L) in males and <50 mg/dL (1.29 mmol/L) in females; (3) raised blood pressure: SBP ≥130 or diastolic BP ≥85 mmHg; or (4) raised fasting plasma glucose (FPG) ≥100 mg/dL (5.6 mmol/L). Dyslipidemia was defined as total high-density lipoprotein <1.04 mmol/L for men and <1.3 for women, and/or triglycerides (TG) ≥1.70 mmol/L, according to the IDF definition (36).

### Sample size calculation

It was assumed that a standard deviation of SBP and DBP were about 14 and 8 mmHg, respectively. One hundred forty-three adults from a representative sample would be sufficient to provide an estimate of SBP and DBP of a population with 95% CI around the mean of about ±120. A large sample size up to 260 was required when precise measures including TG, cholesterol, blood glucose, were measured. As there were 550 adults were recruited into the study, the sample size is adequate for the current study.

### Ethics approval

Ethics approval for the research was obtained from Griffith University the Ethics Committee and the Changshu Centre for Disease Control and Prevention Research Board.

### Statistical analysis

Dietary patterns were derived from the factor analysis by adding all items that loaded highly on each factor. Pearson correlations were used to assess the independence of factors by analyzing the correction level between the pattern scores. Dietary pattern scores were categorized into tertiles with high, medium, and low levels to provide sensitive indicators of the food intake level to assess the association of a food pattern intake to the metabolic factors. Associations between dietary patterns, socio-demographic factors and food habits were investigated using chi-square analysis. Linear trends across tertiles of dietary pattern scores were calculated using a univariate linear modeling method. Means (and standard errors) of BMI, WC, SBP, and DBP were calculated according to tertiles of dietary pattern scores using the univariate linear modeling method and adjusted for age and sex. Only covariates associated with both the dietary patterns and health outcomes (income and exercise) were included in the adjusted models. The level of significance for *p*-values was set at 0.05 for all bivariate analyses and adjusted *p*-value for univariate linear modeling method.

## Results

Statistical assumption has been checked for all categorical variables for adequate cell number for Chi-Square test and continuous variables for Univariate Analysis of Variances. For all categorical variables, they all had adequate cell number to meet the requirement of Chi-Square tests. For all continuous variables, they all had normal distribution with skewness and Kurtosis test within −3 and +3 range.

### Sample characteristics

Table 1 shows, at baseline (*n* = 550), 58.6% of participants had “normal” weight, 34.3% were overweight, and 7% were obese. The prevalence of hypertension was 35.3%. The prevalence of being overweight, obesity, and hypertension was similar to Chinese prevalence rates among older adults (37). In terms of age, 68.3% of participants were in the 50–64 age group, and 31.7% were aged 65–88. All participants were from a Han background. In terms of education level, 42.1% had a low level of primary school education, 40.1% had senior high school level, and 17.7% had a diploma or bachelor level qualification. Most participants were married (89.4%) and retired (95.7%). Nearly half (44.4%) of the participants had an income level of <20,000 Chinese Yuan (RMB) per year, 37.1% had income between RMB 20,000–39,999 per year, and only 18.5% had an income over RMB 40,000 per year. No differences were found between participants who participated in the biomedical assessment and those who did not in the demographic variables of age, gender, marital status, education, but there was a difference in income.

**Table 1:** **Demographic characteristics. of participants**.

Variables	Sample who did not participate biomedical tests *N* (%) (*n* = 200)	Study sample *N* (%) (*n* = 550)	χ^2^	*p*
Age
50–64	136 (68.3)	122 (63.5)	2.69	0.11
65–88	64 (31.7)	70 (36.5)	
Gender
Male	39 (19.6)	154 (78.2)	0.85	0.36
Female	161 (80.4)	43 (21.8)	
Education
Primary schools	84 (42.1)	61 (31.0)	2.10	0.09
Senior high schools	80 (40.1)	91 (46.2)	
TAFE and diploma	27 (13.7)	29 (14.7)	
Bachelor and above	9 (4.0)	16 (8.1)	
Occupation
Retired	191 (95.7)	185 (93.9)	2.57	0.11
Others	9 (4.3)	12 (6.1)	
Marital status
Married	178 (89.4)	171 (87.2)	1.54	0.46
Widowed	14 (6.7)	15 (7.7)	
Never married/divorced/separated	8 (3.9)	10 (5.1)	
Income
<20,000 RMB	89 (44.4)	75 (38.9)	**10.06**	**0.01**
20,000–39,999 RMB	74 (37.1)	68 (35.2)	
40,000 and more	37 (18.5)	50 (25.9)	
BMI
Normal	116 (57.8)	309 (58.6)	0.57	0.75
Overweight	79 (39.7)	181 (34.3)	
Obesity	5 (2.5)	37 (7.0)	
Hypertension
Normal	129 (64.3)	358 (64.7)	0.05	0.91
Hypertension	71 (35.6)	195 (35.3)	

*Bold indicates *p* <0.01*.

Table 2 demonstrates associations of dietary patterns with socio-demographic and behavioral characteristics. Only the soybeans, grains, and flour food pattern showed an association with income level; the high-income group was more likely to have a high intake for this pattern. Only the soybean, grains, and flour food pattern was associated with exercise; more hours of light exercise were evident in participants with this pattern. Age, gender, education level, marital status, and alcohol intake were not related to food intake patterns.

**Table 2:** **Association between diet pattern and socioeconomic factors**.

	Traditional food			Fast and processed food			Soybean, grains, and flour food			Animal liver and other animal meats		
	T1 (*N* = 89)	T2 (*N* = 185)	T3 (*N* = 196)	T1 (*N* = 216)	T2 (*N* = 141)	T3 (*N* = 189)	T1 (*N* = 75)	T2 (*N* = 191)	T3 (*N* = 182)	T1 (*N* = 238)	T2 (*N* = 164)	T3 (*N* = 144)
Age
50–64 (%)	19.0	37.9	43.1	40.0	25.4	34.6	18.4	41.6	40.0	44.5	28.8	26.7
(65 (%)	18.9	42.7	38.5	38.4	26.8	34.6	13.0	44.9	42.0	41.5	32.9	25.6
Gender
Female (%)	19.2	37.9	42.9	38.8	25.1	36.1	15.1	42.9	42.0	44.6	29.4	26.0
Male (%)	18.1	45.7	36.2	42.3	27.9	29.8	22.7	42.0	35.2	38.5	34.6	26.9
Qualification
Primary school (%)	20.4	35.1	44.5	41.8	23.5	34.7	20.2	40.4	39.4	47.8	27.9	24.3
Secondary school (%)	17.6	45.1	37.4	39.1	26.6	34.3	14.0	47.2	38.8	41.5	30.0	28.5
Bachelor (%)	18.7	38.7	42.7	32.1	27.4	40.5	14.3	40.0	45.7	33.3	40.5	26.2
Income (in RMB)
<20,000 (%)	20.0	39.5	40.5	41.3	24.7	34.0	19.1	45/6	34.5	44.1	31.2	24.7
20,000–39,999 (%)	18.6	38.4	43.0	36.3	26.9	36.8	14.5	42.8	42.8	41.3	28.4	30.3
40,000 or more (%)	14.9	43.0	44.6	36.1	26.5	37.3	10.1	34.8	**55.1**	39.8	37.3	22.9
Marital status
Married (%)	19.0	38.0	43.0	39.7	24.4	35.9	17.3	44.0	38.8	44.0	30.2	25.8
Widowed (%)	18.2	57.6	24.2	29.4	41.2	29.4	15.2	33.3	51.5	29.4	35.3	35.3
Others (%)	20.0	40.0	40.0	47.4	31.6	21.1	10.0	30.8	69.2	52.6	26.3	21.1
Exercise *M* (SD)
Moderate activity (hour)	1.76 (1.21)	1.67 (1.07)	1.79 (1.28)	1.77 (1.20)	1.52 (0.99)	1.72 (1.14)	1.82 (1.19)	1.69 (1.16)	1.68 (1.19)	1.73 (1.21)	1.75 (1.16)	1.54 (0.96)
Sleep (hour)	7.46 (1.26)	7.57 (1.07)	7.65 (1.10)	7.74 (1.11)	7.63 (1.21)	7.74 (1.04)	7.52 (1.27)	7.64 (1.19)	7.60 (1.02)	7.72 (1.00)	7.53 (1.27)	7.70 (1.13)
Extreme light exercise (hour)	2.83 (1.72)	2.88 (1.70)	3.07 (1.69)	3.22 (1.96)	2.78 (1.70)	3.07 (1.73)	2.89 (1.85)	2.99 (1.75)	2.99 (1.54)	3.19 (1.68)	3.22 (2.00)	2.81 (1.57)
Light exercise (hour)	2.25 (1.13)	2.29 (1.19)	2.52 (1.31)	2.56 (1.44)	2.38 (1.25)	2.34 (1.11)	2.22 (1.05)	2.45 (1.13)	**2.50 (1.42)^a^**	2.36 (1.23)	2.31 (1.17)	2.34 (1.13)
Alcohol intake
No (%)	18.8	39.8	41.4	40.5	24.5	35.0	17.6	42.0	40.4	45.0	29.6	25.4
Yes (%)	20.3	37.8	41.9	32.9	32.9	34.2	11.6	47.8	40.6	34.2	35.4	30.4

*^a^ Bold indicates statistical significance, p < 0.05*.

Table 3 demonstrates the association between dietary pattern and biomarkers. Income and light level exercise were associated with the soybeans, grains and flour dietary pattern; therefore, only these two confounders were included in the adjusted models in the analysis of the relationship between dietary pattern and health outcomes (Table 3). The level of significance for *p*-values was set at 0.02 for the analysis of the relationship between soybeans, grains, and flour dietary pattern and biomarkers due to the multiplicity of the analysis. The traditional food pattern that included fruit, vegetable, rice, and fish was significantly associated with SBP (*p* = 0.025). Among the three levels of food intake scores, participants with a moderate level of traditional food intake had lower SBP and cholesterol levels than those with a high level of traditional food intake.

**Table 3:** **Relationship between food pattern and blood pressure, BMI, and metabolic factors using a univariate general linear model**.

	T	Traditional food				Processed food				Bean, grains, and flour food				Animal liver and other animals meat			
		*N*	Mean	*p*	*Post hoc*	*N*	Mean	*p*	*Post hoc*	*N*	Mean	*p*	*Post hoc*	*N*	Mean	*p*	*Post hoc*
Systolic blood pressure (BP)	T1	139	136.89 (13.92)	**0.03**	T2 < T3*	92	131.60 (15.58)	0.14		169	130.83 (16.36)	0.46		150	129.90 (15.90)	0.76	
	T2	168	130.21 (14.84)			121	132.31 (16.17)			138	131.43 (15.84)			135	132.52 (15.98)	
	T3	170	135.53 (16.61)			123	128.40 (16.48)			151	129.19 (15.67)			129	128.57 (15.77)	
Diastolic blood pressure	T1	139	81.72 (7.17)	0.10		92	81.92 (8.20)	0.65		169	82.20 (8.33)	0.76		150	83.28 (7.84)	0.06	
	T2	168	81.38 (8.33)			121	82.76 (7.80)			138	82.60 (7.98)			135	83.27 (8.16)	
	T3	170	83.14 (8.28)			123	81.89 (8.38)			151	81.91 (7.82)			129	80.94 (8.13)	
Serum cholesterol	T1	147	4.31 (0.84)	**0.02**	T2 < T3*	97	4.60 (1.38)	0.52		180	4.74 (1.40)	0.12		150	4.68 (1.26)	0.53	
	T2	182	4.20 (0.89)			126	4.99 (1.99)			141	5.08 (3.94)			135	4.65 (1.24)	
	T3	181	4.48 (0.97)			137	4.70 (4.18)			168	4.50 (1.60)			129	5.04 (4.55)	
High-density lipoprotein cholesterol (mmol/L)	T1	147	1.51 (0.56)	0.51		97	1.51 (0.56)	0.50		180	1.56 (0.59)	0.47		150	1.55 (0.50)	0.94	
	T2	182	1.54 (0.56)			126	1.59 (0.51)			141	1.59 (0.48)			135	1.53 (0.54)	
	T3	181	1.58 (0.61)			137	1.52 (0.64)			168	1.51 (0.64)			129	1.53 (0.66)	
Triglycerides (mmol/L)	T1	147	1.62 (1.24)	0.81		97	1.65 (1.23)	0.10		180	1.95 (1.78)	**0.00**	T2 < T1*	150	1.71 (1.33)	0.65	
	T2	182	1.66 (1.30)			126	1.94 (1.77)			141	1.51 (0.80)		T3 < T1**	135	1.75 (1.50)	
	T3	181	1.71 (1.53)			137	1.56 (1.28)			168	1.48 (1.15)			129	1.58 (1.35)	
Low-density lipoprotein (mmol/L)	T1	147	2.81 (1.74)	0.32		97	2.89 (0.67)	0.74		180	2.65 (1.02)	0.08		150	2.63 (0.92)	0.93	
	T2	182	2.62 (1.00)			126	2.90 (0.68)			141	2.88 (1.64)			135	2.64 (0.94)	
	T3	181	2.63 (0.97)			137	2.97 (0.75)			168	2.57 (1.09)			129	2.68 (1.06)	
Fasting glucose (mmol/L)	T1	147	5.62 (1.65)	0.54		97	5.33 (0.16)	0.81		180	5.79 (1.84)	**0.03**	T3 < T1*	150	5.72 (1.70)	0.25	
	T2	182	5.52 (1.65)			126	5.20 (0.14)			141	5.77 (1.27)			135	5.68 (1.72)	
	T3	181	5.72 (1.91)			137	5.07 (0.35)			168	5.35 (1.96)			129	5.36 (1.88)	
Serum creatinine	T1	147	59.96 (18.52)	0.38		97	60.72 (19.46)	0.05		180	60.88 (21.06)	0.09		150	61.33 (17.26)	0.49	
	T2	182	61.22 (20.90)			126	62.76 (17.85)			141	61.81 (14.35)			135	61.22 (17.94)	
	T3	181	58.39 (18.27)			137	60.05 (20.83)			168	57.34 (20.57)			129	58.52 (23.61)	
Waist (cm)	T1	130	77.29 (7.85)	0.34		88	78.26 (8.35)	0.07		162	77.61 (8.53)	**0.04**	T3 < T1**	150	77.31 (8.75)	0.24	
	T2	164	75.92 (8.03)			115	75.94 (7.48)			131	76.19 (7.88)			135	75.44 (7.58)	
	T3	165	76.45 (7.89)			123	75.93 (8.09)			147	75.39 (7.00)			129	76.44 (7.25)	
Hips (cm)	T1	129	93.39 (7.01)	0.28		88	94.15 (7.31)	0.28		161	93.42 (6.69)	0.56		150	92.97 (7.13)	1.00	
	T2	164	92.23 (6.38)			115	92.34 (5.95)			130	92.44 (6.28)			135	92.92 (10.75)	
	T3	164	93.45 (9.33)			122	93.46 (10.36)			147	93.02 (9.77)			129	93.02 (5.87)	
Waist hip ratio	T1	129	0.83 (0.05)	0.53		88	0.83 (0.05)	0.13		161	0.83 (0.05)	**0.03**	T3 < T1*	150	0.83 (0.05)	0.16	
	T2	164	0.82 (0.05)			115	0.82 (0.05)			130	0.82 (0.05)			135	0.82 (0.07)	
	T3	164	0.82 (0.06)			122	0.81 (0.07)			147	0.81 (0.06)			129	0.82 (0.05)	
Body mass index	T1	130	23.82 (3.18)	0.12		88	24.04 (3.18)	0.12		163	23.68 (3.12)	0.37		150	24.31 (3.83)	**0.02**	**T3 > T1**
	T2	165	23.08 (2.98)			116	23.31 (2.87)			132	23.21 (3.04)			135	24.44 (3.47)	
	T3	166	23.43 (2.88)			123	23.23 (2.99)			147	23.32 (2.89)			129	25.28 (3.64)	

Incomes were adjusted in the univariate ANOVA analysis in the bean, grains, and flour food pattern. Bold indicates statistical significance, *p < 0.05; **p < 0.01; ***p < 0.001.

The processed and fast food pattern was not significantly associated with any of the biomedical markers. The soybeans, grains, and flour food pattern was associated with WC (*p* = 0.04) and waist–hip ratio (*p* = 0.03), blood glucose (*p* = 0.03), and statistically significantly associated with triglycerides. Participants with a high level of soybeans, grains, and flour food intake had lower blood glucose (*p* = 0.03), WC (*p* < 0.001), and waist–hip ratio (*p* = 0.03) than those with a low level of this food intake pattern. High intake levels of milk products, animal liver, and other animal meats were associated with increased BMI levels.

## Discussion

This study is the first to explore the association between dietary patterns and health outcomes in a population-based sample of Chinese older adults. The findings demonstrate that dietary patterns of Chinese older adults are associated with health outcomes. A moderate intake of “traditional Chinese food” is associated with reduced levels of SBP and cholesterol. A high intake of soybeans, grains, and flour is associated with a reduced level of metabolic factors, and a high intake of milk products, animal liver, and other animal meats is associated with increased BMI levels. There is no relationship between processed food and any of the cardiovascular disease risk factors. The hypothesis of a relationship between dietary pattern and cardiovascular risk is supported in three out of four diets identified in our factor analysis. It is difficult to compare these findings with earlier studies conducted in Western countries because Chinese older adults have substantially different food intake patterns. Regardless, the patterns identified demonstrate some similarities with previous work among older adults. For example, the fruit, vegetables, eggs, pork, and fish pattern as a “traditional” pattern has nutrient content similar to “vegetables, fruit, beef, lamb, fish, and grains” in other countries. The processed and fast food pattern is similar to the “Western food” pattern that includes meat pies, processed meats, pizza, chips, hamburgers, white bread, and sugar, while the soybeans, grains, and flour pattern is similar to the “modern” food pattern. Finally, the dairy, animal liver, and other animal meat food pattern is similar to the traditional Western food pattern (38).

The validity of the dietary patterns was assessed by examining the association between food patterns and biomarkers. Several methodological issues strengthen confidence in the questionnaire. The screening tool was developed based on 24-h food intake data in the previous month and is quantitative, thus minimizing errors in memory and computation. Notable correlations with biomarkers of nutritional status provide support for the questionnaire, because measurement error in the questionnaire is not likely to be correlated to measurement error in the assessment of biomarkers. Bailey et al. (23) and van Staveren et al. (39) have indicated favorable intakes of micronutrients and biomarkers among older adults who consume foods rich in fruit and grains. Although pattern differences across groups are likely, the argument for generalizability is that the patterns detected using the questionnaire were similar to those found in more diverse samples, with significantly more comprehensive dietary assessment methods.

This study found that only the soybean, grains, and flour dietary pattern was associated with measures of income, which is consistent with research carried out in various populations using different indices (13, 14, 31). The general observed socioeconomic nutritional gradient can be mediated by food costs, meaning that the lowest cost diets, mainly consumed by the lowest socioeconomic positions, are generally unhealthy. People who consume higher levels of soybean products, grains, and flour food reported higher incomes, suggesting that these foods are viewed by this group as high quality and affordable.

Participants with moderate intakes of traditional Chinese food, including the fruit, vegetables, pork, chicken, rice, and fish pattern, had lower SBP and serum cholesterol levels, but no other biomarkers. No previously published literature explains this outcome, but it may be that the nutritional characteristics of the traditional Chinese diet (for instance) positively affect blood pressure levels. These characteristics include moderate levels of fruit, vegetables, and protein, possibly leading to dietary-induced lowered blood pressure level (17). The prevalence of hypertension and stroke is high for older Chinese adults (17), and the positive association between healthy food intake and blood pressure is well documented. Considering that the moderate intake of traditional Chinese foods is the dominant food pattern, this food intake pattern may be one of the causes of decreased blood pressure levels. Processed and fast food was not related to any of the biomedical markers. It is speculated that the low intake of processed and fast food may account for its minimal-level effect on metabolism, and may account for the fact that the prevalence of coronary heart disease among Chinese is lower than it is for Caucasians (40).

The soybean, grains, and flour food dietary pattern, which was correlated with a lower WC and waist–hip ratio, has also been associated with several biomarkers for reduced cardiovascular risk factors, including a reduced level of serum lipoproteins and blood glucose. The high consumption of soybean products may contain more phytoestrogen, which has protective effects against heart disease (13). The finding that consumers of the soybean, grains, and flour pattern had lower blood glucose, triglycerides, WC, and waist–hip ratios is consistent with previous research (41) and, therefore, provides additional evidence that a dietary intake of these foods may reduce the risk of obesity, metabolic syndrome, and diabetes in Chinese older adults.

Components of the milk products, animal food dietary pattern included animal liver and other animal foods. Higher scores on this pattern likely accompany greater consumption of fat, especially saturated fat, which may increase the risk of diabetes, metabolic syndrome, and hypertension (16, 42). The Western dietary pattern is characterized by frequent red meat intake, which increases the risk of cardiovascular disease (16, 42). Consistent with these findings, the present study found that the milk products and animal food dietary pattern was significantly associated with an increased level of BMI. Dietary habit change involving less consumption of other animal meats may offer avenues for minimizing high BMI levels.

Our detailed analysis of dietary patterns reflects intakes of nutrients important for the prevention of factors associated with high SBP, metabolic abnormalities, and cardiovascular disease-related biomedical risk factors. This highlights that the dietary patterns measure underlying differences in nutrient intakes, capture biologically relevant exposures, and describe eating patterns. Overall, Chinese foods have less fat than European diets. Consuming a variety of food, including soybeans, grains, and flour food, milk products, and a moderate level of other animal meats therefore may be complementary to the moderate-level traditional food intake in China. This is evident from the association of biomarkers with the two main food patterns that are protective against high blood pressure, high metabolic abnormality level, and high levels of WC and waist–hip ratio.

The moderate “traditional Chinese food” intake is related to a reduced chance of having high blood pressure compared with that of the high-level “traditional Chinese food” intake. Intake levels in particular food patterns are related to different metabolic outcomes as follows: (a) a high level of soybean product, grain, and flour food pattern is related to a reduced chance of being overweight or obese, and having a metabolic abnormality and glucose intolerance; and (b) a high level of dairy and other cooked animal meats is related to an increased risk of being overweight and obesity.

The results arising from these food intake patterns suggest that moderate intakes of “traditional Chinese food,” high-level intakes of soybean products and grains, and low-level intakes of milk products and animal meats are protective against metabolic syndrome, high blood pressure, elevated triglycerides, and obesity, all of which are key risk factors for cardiovascular disease and glucose intolerance, a key risk factor for Type 2 diabetes.

A limitation of this study is the high proportion of older adults (*n* = 200; 26.7%) who did not complete a biomarker test and were thus excluded from the analysis; however, there were no differences between people who participated in the study and those who did not participate. In addition, the effect of a moderate intake level of traditional Chinese food is only speculative due to the nature of the cross-sectional study data and further nutrient data analysis is required to confirm this result. Further study using prospective cohort or intervention study should be used to confirm the association between dietary patterns and blood pressure and metabolic factors. Although the sample size is large with 550 people consented to the study, no all participants completed questions and biomedical assessments, so the number of participants varied for assessment items. The bias caused by these missing data should be taken into consideration when the conclusion is made and when study is generalized to other regions in China.

In conclusion, the present results indicate that a dietary pattern characterized by frequent consumption of a moderate level of “traditional Chinese food,” high-level intake of soybean products and dairy products, combined with a low intake of animal meat products, may be associated with a reduced risk of impaired glucose tolerance, reduced SBP in Chinese older adults and BMI. Although these findings need to be confirmed by prospective studies, interventions to change dietary patterns should be considered to decrease cardiovascular disease risk among Chinese older adults, whose consumption of dairy food and fruit is currently low.

### Relevance to clinical practice

We recommend that strategies targeting relevant dietary intake factors be implemented in the population of adults aged 50 and above in China to manage cardiovascular disease risk factors. Health professionals such as nurses may be best suited to providing such interventions given their integral role of providing advice to people with unhealthy food intake. Cardiovascular disease management should be implemented in community medical centers close to the population, and focus service delivery on measures to assist patients to consume a moderate level of “traditional Chinese food,” a high level of soybean products and dairy products, and a low intake of animal meat products.

## Authors Contribution

Dr. Jing Sun: conceptualized and designed the study, did field work, prepared and drafted manuscript paper, analyzed data, and revised the manuscript. Professor Nicholas Buys: designed the study, reviewed, and revised the manuscript. Dr. Shuying Shen: designed the food frequency questionnaire, and proofed the manuscript.

## Conflict of Interest Statement

The authors declare that the research was conducted in the absence of any commercial or financial relationships that could be construed as a potential conflict of interest.
